# Pathogenesis of Two Western Mediterranean West Nile Virus Lineage 1 Isolates in Experimentally Infected Red-Legged Partridges (*Alectoris rufa*)

**DOI:** 10.3390/pathogens10060748

**Published:** 2021-06-13

**Authors:** Virginia Gamino, Elisa Pérez-Ramírez, Ana Valeria Gutiérrez-Guzmán, Elena Sotelo, Francisco Llorente, Miguel Ángel Jiménez-Clavero, Ursula Höfle

**Affiliations:** 1 Grupo SaBio (Sanidad y Biotecnología), Instituto de Investigación en Recursos Cinegéticos (IREC) (CSIC-UCLM-JCCM), 13071 Ciudad Real, Spain; gamino.virginia@gmail.com (V.G.); valerivet@hotmail.com (A.V.G.-G.); 2 Centro de Investigación en Sanidad Animal (CISA) del Instituto Nacional de Investigación y Tecnología Agraria y Alimentaria (INIA-CSIC), 28130 Madrid, Spain; elisaperezramirez@gmail.com (E.P.-R.); e.sotelo@zendal.com (E.S.); dgracia@inia.es (F.L.); majimenez@inia.es (M.Á.J.-C.); 3 Centro de Investigación Biomédica en Red de Epidemiologia y Salud Pública (CIBERESP), 28029 Madrid, Spain

**Keywords:** West Nile virus lineage 1, pathogenesis, neurovirulence, red-legged partridge, antigen distribution, inflammatory reaction

## Abstract

West Nile virus (WNV) is the most widespread flavivirus in the world with a wide vertebrate host range. Its geographic expansion and activity continue to increase with important human and equine outbreaks and local bird mortality. In a previous experiment, we demonstrated the susceptibility of 7-week-old red-legged partridges (*Alectoris rufa*) to Mediterranean WNV isolates Morocco/2003 and Spain/2007, which varied in virulence for this gallinaceous species. Here we study the pathogenesis of the infection with these two strains to explain the different course of infection and mortality. Day six post-inoculation was critical in the course of infection, with the highest viral load in tissues, the most widespread virus antigen, and more severe lesions. The most affected organs were the heart, liver, and spleen. Comparing infections with Morocco/2003 and Spain/2007, differences were observed in the viral load, virus antigen distribution, and lesion nature and severity. A more acute and marked inflammatory reaction (characterized by participation of microglia and CD3+ T cells) as well as neuronal necrosis in the brain were observed in partridges infected with Morocco/2003 as compared to those infected with Spain/2007. This suggests a higher neurovirulence of Morocco/2003, probably related to one or more specific molecular determinants of virulence different from Spain/2007.

## 1. Introduction

West Nile virus (WNV) is an arthropod-borne flavivirus whose natural cycle involves bird hosts and mosquito vectors, with horses and humans as accidental or dead-end hosts [1]. Currently, it is considered one of the most widely distributed arboviruses in the world, causing numerous human, equine, and bird outbreaks and mortalities, both in the Old and New World [2,3]. In the Mediterranean basin, WNV activity is continuously increasing, and has been associated with several outbreaks affecting mainly humans and horses [4,5]. In this area, avian mortality due to WNV lineage 1 (L1) has been sporadic, while lineage 2 (L2) WNV has been responsible for significant outbreaks in central Europe and in magpies in Italy and Greece [6,7,8,9,10,11,12,13]. Nevertheless, under laboratory conditions in experimental infections, at least some Mediterranean L1 WNV strains have proven to be pathogenic for European wild bird species [14,15,16,17,18].

In an experimental study we demonstrated that the red-legged partridge (*Alectoris rufa*), a Mediterranean endemic gallinaceous bird species [19], is susceptible to the infection with two lineage 1 Western Mediterranean WNV strains, Morocco/2003 [20] and Spain/2007 [17]. However, the virulence of both strains differed, with 70% mortality in Morocco/2003-infected partridges as compared to 30% mortality in Spain/2007-infected birds, corroborating the results observed in a previous study in a mouse model [21]. 

The objective of the present work was to study the pathogenesis of WNV in the partridges in the mentioned experimental infection in detail to explain the different infection course and mortality observed between the two WNV strains. For this purpose, we compared the viral load, dynamics of virus appearance, and distribution and severity of microscopic lesions in different tissues. Additionally, we studied the dynamics of inflammatory cell activation and recruitment into the central nervous system (CNS) of the infected partridges. The objective was twofold: on one hand, to determine the evolution of the immune response in the CNS of the avian host, as this information is scarce for birds; and on the other hand, to determine differences in the response induced by the two WNV strains, as this could be considered a virulence marker [22].

## 2. Results

### 2.1. Clinical Signs and Gross Pathology

As described in our previous study [17], clinical signs included loss of appetite, ruffled feathers, paralysis, and lack of responsiveness. These started earlier (4 vs. 5 dpi) and were more severe in partridges infected with Morocco/2003. Macroscopic lesions were observed in all euthanized animals from 3 dpi on. The most affected organs were the heart, spleen, liver, and kidney. While the heart and kidney showed lesions from 3 dpi, the spleen and liver were not affected until 6 dpi. Main macroscopic lesions were pallor of the myocardium and hepatic, splenic, and renal parenchyma, as well as the presence of diffuse petechiae. At 14 dpi, congestion of the kidney and lung were also observed.

### 2.2. Virus Genome Detection

All tissue samples collected from euthanized animals at 3 and 6 dpi tested positive by RRT-PCR. The lowest Ct values (i.e., highest viral loads) were reached at 6 dpi (Table 1). The viral load in tissues, estimated using Ct values, was similar in Morocco/2003- and Spain/2007-infected partridges (or slightly higher for Morocco/2003) (Table 1). At 3 and 6 dpi, the highest viral loads were found in the spleen, kidney, and heart (Table 1). High amounts of viral RNA were also detected in feather pulps collected at 6 dpi (Table 1). At 14 dpi, viral loads were low in most cases, and several tissues were negative in the Spain/2007-infected partridges (Table 1). 

### 2.3. Histopathology

**Figure 1:**
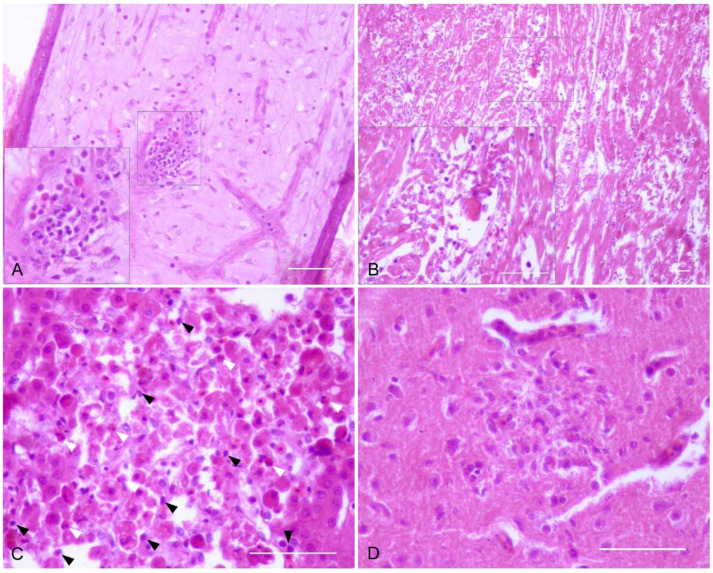
Microscopic lesions in tissues of experimentally WNV-infected red-legged partridges. (**A**) Feather follicle; partridge inoculated with Morocco/2003, 14 dpi. Multifocal infiltration of inflammatory cells in the feather pulp. Inset: detail of an inflammatory nodule composed of lymphocytes, macrophages, and granulocytes. (**B**) Heart; partridge inoculated with Spain/2007, 6 dpi. Diffuse and marked necrosis and degeneration of cardiac myofibers, and infiltration of inflammatory cells. Inset: detail of necrotic cardiac myofibers which show degeneration, fragmentation, and accumulation of hyaline material in the cytoplasm. Infiltration of mononuclear inflammatory cells is also observed. (**C**) Liver; partridge inoculated with Morocco/2003, 6 dpi. Necrosis of hepatocytes characterized by cellular detachment, lysis of the cytoplasm and pyknosis and fragmentation of the nucleus. There is also a mild infiltration of lymphocytes and plasma cells (black arrowheads) and heterophils (white arrowheads). (**D**) Cerebrum; partridge inoculated with Morocco/2003, 6 dpi. Focal necrosis with degeneration of the neuropil and infiltration of lymphocytic and glial cells. Scale bars = 100 µm.

Microscopic lesions appeared as early as 3 dpi but were more severe and widespread at 6 dpi (Table 2). At that time, the most affected tissues were the heart, lung, spleen, liver, and bursa of Fabricius (Table 2). The main microscopic finding was the presence of inflammatory infiltrates from 3 dpi onwards. These were mainly composed of lymphocytes, macrophages, and plasma cells. Heterophils were also detected in the liver, spleen, and intestine and, to a lesser extent, in the heart, feather follicles (Figure 1A), and skin of both groups. Cellular degeneration and/or necrosis were especially severe in the heart and liver at 6 dpi and in the kidney at 14 dpi (Figure 1B–C, Table 2). On those days, the liver and spleen showed hemosiderosis. In the brain, endothelial cell swelling was observed throughout the experiment in both groups; however, the development of a non-suppurative encephalitis and neuronal necrosis occurred earlier in partridges infected with Morocco/2003 (Figure 1D, Table 2).

**Figure 2:**
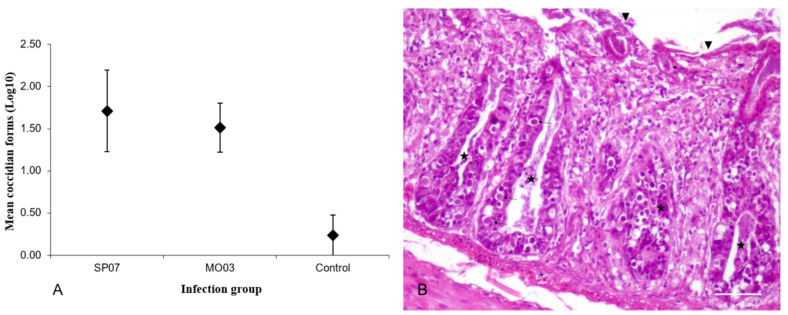
Coccidiosis in the large intestine of WNV-infected and control red-legged partridges. (**A**) Graph showing the mean numbers ± standard deviation of log transformed nos. of coccidian forms in transverse sections of the large intestine at the level of cecal tonsils in red-legged partridges experimentally infected with WNV Morocco/2003 (MO03) and Spain/2007 (SP07) strains. (**B**) Cecum; partridge inoculated with Morocco/2003, 6 dpi. Presence of gametes and coccidian oocysts in the epithelium of the mucosa and crypts (arrows). There is also inflammation in the lamina propria and severe necrosis of the epithelium of both the mucosa (arrowheads) and crypts (stars). Scale bar = 100 µm.

As an incidental finding, there were coccidian oocysts and gametes in the enterocytes of the large intestine. These parasites were much more numerous in WNV-infected birds as compared to control individuals (Figure 2A), especially in partridges euthanized at 6 dpi and in those infected with Morocco/2003. In fact, one bird of this group showed an associated severe necrosis of the epithelium of intestinal mucosa and crypts (Figure 2B).

### 2.4. Virus Antigen

**Figure 3:**
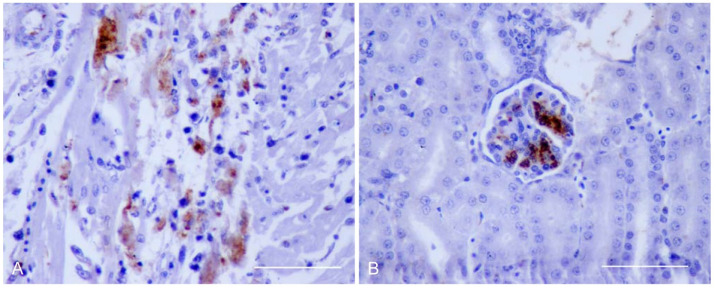
Detection of WNV antigen by immunohistochemistry in tissues of experimentally-infected red-legged partridges. (**A**) Heart; partridge inoculated with Spain/2007, 6 dpi. WNV antigen in the cytoplasm of cardiac myofibers. (**B**) Kidney; partridge inoculated with Morocco/2003, 6 dpi. WNV antigen in the cytoplasm of glomerular mesangial cells. Scale bars = 100 µm.

Virus antigen staining was mild to moderate in most cases. Immunopositivity was more widespread at 6 dpi and in the tissues of partridges infected with Morocco/2003 (Table 3), consistent with RRT-PCR results. At 3 dpi, WNV antigen was detected in macrophages in spleen and inflammatory cells and myofibers of the heart of both groups. In Morocco/2003-infected partridges there was also a mild immunostaining in tubular epithelial cells of the kidney, isolated acinar cells of the pancreas, and a small group of inflammatory cells in the cecal tonsils. At 6 dpi, the WNV antigen was stained in inflammatory cells in the lung, heart, spleen, kidney, and pancreas. It was also detected in cardiac myofibers (Figure 3A), glomerular mesangial cells (Figure 3B), and tubular epithelial cells of the kidney, acinar cells of the pancreas, as well as in cells of the crypts and myofibers of the muscularis externa of the large intestine. Only in birds infected with Morocco/2003 was the WNV antigen evidenced in smooth muscle cells of the splenic vessels, hepatocytes, and Kupffer cells of the liver, as well as smooth muscle cells of the vascular wall of one vessel and several myofibers of the muscularis externa of the duodenum. At 14 dpi, IHC was negative in all examined tissues (Table 3).

### 2.5. Inflammatory Cells in the Brain

#### 2.5.1. Microglia Activation/Macrophage Infiltration

**Figure 4:**
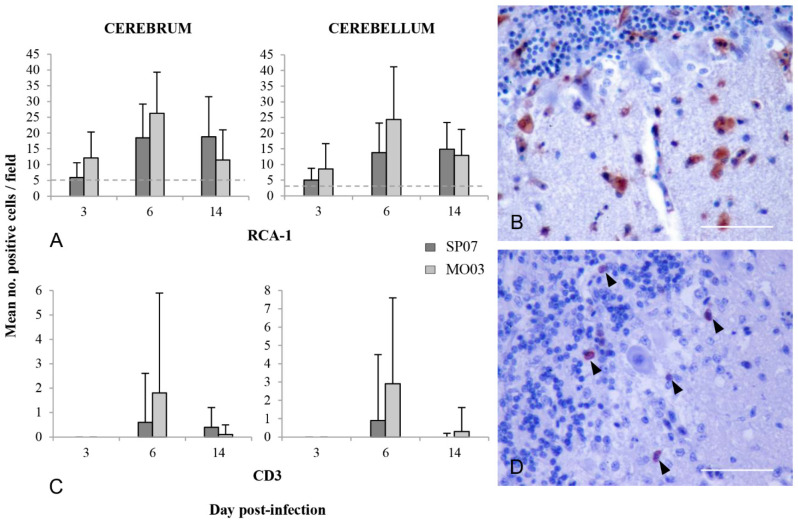
Characterization of inflammatory cell reaction in the brain of red-legged partridges experimentally infected with WNV Morocco/2003 (MO03) and Spain/2007 (SP07) strains. (**A**) Semi-quantitative analysis of the number of RCA-1+ microglial cells/macrophages in the cerebrum and cerebellum at different days post-inoculation (dpi). Bars represent the mean of stained cells in 30 randomly selected fields (at 400x magnification) ± standard deviation. The horizontal dashed line represents the mean of stained cells in the cerebrum and cerebellum of a non-WNV-infected partridge. (**B**) Cerebellum; partridge inoculated with Morocco/2003, 6 dpi. RCA-1 positive staining in the cytoplasm of phagocytic foamy macrophages in the molecular layer. Scale bar = 100 µm. (**C**) Semi-quantitative analysis of the number of CD3+ T cells in the cerebrum and cerebellum at different dpi. Bars represent the mean of stained cells in 30 randomly selected fields (at 400x magnification) ± standard deviation. (**D**) Cerebellum; partridge inoculated with Morocco/2003, 6 dpi. CD3 positive staining in T cells located near the Purkinje cell layer (arrowheads). Scale bar = 100 µm.

At 3 dpi, a mild reaction of microglial cells was observed in Morocco/2003-infected partridges. Nevertheless, it was at 6 dpi when these cells were more active in both groups, especially in birds infected with Morocco/2003 (Figure 4A). At 14 dpi, while the activity of microglial cells decreased in the aforementioned group, these remained very active in Spain/2007-infected individuals (Figure 4A).

In the cerebrum, at 3 dpi, RCA-1+ ramified cells were diffusely distributed in the parenchyma. At that time, there was also the mild presence of amoeboid (large soma and short, thick cellular processes) and rounded cells (corresponding both to activated microglia and macrophages). From 6 dpi onwards, most microglia changed to an activated amoeboid morphology and increased the presence of microglia/macrophage nodules, which in many cases surrounded neurons and vessels. RCA-1+ cells were especially abundant in the peripheral pallium and in the region located near the lateral ventricle. In the cerebellum, microglial cell reaction was milder than in the cerebrum (Figure 4A), but the evolution of changes in cellular morphology was similar. These cells were more active in the molecular layer, some of them surrounding Purkinje cells (Figure 4B). At 6 dpi, cell activation was also moderate in the granular layer and white matter, especially in Morocco/2003-infected partridges.

#### 2.5.2. Astrocyte Activation

GFAP+ cells were detected from 3 dpi on, with mild changes in their distribution and relative abundance during the infection course and between infected groups. Compared to a non-infected partridge, there were mild differences in staining distribution and the quantity of stained cells but none in astrocyte morphology or staining intensity.

In the cerebrum, moderate astrocytosis (i.e., increased number of astrocytes) was detected near the lateral ventricle in both groups and in the lamina medularis dorsalis in Morocco/2003-infected birds. In the cerebellum, there was mild astrocytosis in the granular layer, slightly more marked at 6 dpi and in Morocco/2003-infected partridges. In this group, and especially in one bird euthanized at 6 dpi, some GFAP+ fibers invaded the molecular layer and among Purkinje cells. Moderate astrocytosis was also observed in the white matter of Morocco/2003-infected birds.

#### 2.5.3. T Cell Infiltration

CD3+ T cells infiltrated the brain parenchyma after 3 dpi, and at 6 dpi these cells were more numerous, especially in Morocco/2003-infected birds (Figure 4C). At 14 dpi, the number of T cells decreased, more sharply in the Morocco/2003-infected group (Figure 4C). In some cases, brain zones of T cell infiltration corresponded to brain zones of microglia activation and/or macrophage infiltration. Few T cells were detected in meningeal vessels, and only in Morocco/2003-infected partridges.

In the cerebrum, CD3+ T cells were found diffusely distributed, but also forming part of perivascular infiltrates and of inflammatory foci/nodules associated, in some cases, with neuronal necrosis. The peripheral pallium was especially infiltrated by these cells. In the cerebellum, the vast majority of CD3+ T cells were distributed in the molecular layer, in some cases forming part of inflammatory nodules surrounding Purkinje cells (Figure 4D). There was also moderate infiltration in the granular layer and mild infiltration in the white matter.

## 3. Discussion

In this work, we studied differences in the pathogenesis after experimental infection of red-legged partridges with two different Mediterranean L1 WNV isolates, Morocco/2003 and Spain/2007, to elucidate the cause of the different infection course and mortality observed during the experiment and described in a previous work [17].

In both experimental groups, the WNV genome and antigen as well as macroscopic and microscopic lesions were detected as early as 3 dpi. Nevertheless, day 6 post-inoculation (3 days after peak viremia, [17]) can be considered critical in the course of the infection, since at that time, the viral load in most tissues reached the highest levels, the virus antigen was more widespread and abundant, and microscopic lesions were more severe. At 14 dpi, although microscopic lesions were still observed, the low viral loads in organs and the absence of virus antigen detectable by IHC suggest that, by this dpi, the partridges had cleared most of the infecting virus. Nevertheless, potential virus maintenance in some tissues of a low percentage of infected individuals should be considered, as persistent infection has been demonstrated in experimentally and naturally WNV-infected birds [23,24].

Microscopic lesions found in the partridges were consistent with those described in other WNV-infected gallinaceous birds [25,26,27]. Nevertheless, while we found endothelial cell swelling, gliosis, and neuronal necrosis in the brain, CNS lesions were not described in chukar partridges (*Alectoris chukar*) naturally infected with WNV [26,28]. In contrast, in experimentally infected ruffed grouse (*Bonasa umbellus*) who were native from the North American continent, encephalitis of differing severity was present in all, even vaccinated, individuals [29]. Differences in infection conditions, such as the specific virus strain, inoculation dose, and age or bird species, could account for the differences in pathological findings [18,30]. The marked presence of inflammatory infiltrates in feather pulps and skin from 3 dpi onwards was probably associated with the high viral load found by RRT-PCR. WNV has been detected in the skin and feather follicles in naturally and experimentally infected birds [31,32,33], and feather-picking has been suggested as a potential way of horizontal WNV transmission [34]. The exacerbation of coccidian oocysts infestation in the intestines, concurrent with the acute phase of WNV infection (3–6 dpi), suggests a potential WNV-mediated effect on immune function, although more studies are necessary to support this hypothesis. Secondary pathological processes in WNV-infected birds have been documented on numerous occasions [7,13,35,36].

In general, the viral loads detected by RRT-PCR correlated well with the virus antigen detected by IHC and microscopic lesions, with some exceptions such as the feather pulp or the brain. Differences in the results between RRT-PCR and IHC can be explained by their different sensitivity (they detect different viral components), specific areas analyzed, or just by differences in sample collection. In the specific case of the brain, negative results in IHC, despite the mild to moderate neuronal necrosis, could also be explained by the induction of cell injury by the local inflammatory response rather than the direct effect of neuronal virus infection, as has been previously suggested [37,38,39]. However, considering our RRT-PCR results in this tissue, the lower sensitivity of IHC is a more likely explanation. In comparison free-ranging goshawks (*Accipiter gentilis*) infected with lineage 2 WNV predominantly had CNS lesions and abundant WNV antigen detection in foci associated with lesions, in addition to severe myocarditis and lesions in the liver, spleen, and kidney [40]. Additionally, free-living magpies infected with WNV L2 showed severe CNS signs while, in contrast, experimentally infected red-legged partridges, our study species, had only non-specific signs and macroscopic lesions similar as those observed in WNV L1-infected birds, although in both studies no histopathologic descriptions were included [12,41]. This suggests, on the one hand, that bird-virulent WNV L2 strains may be more neurovirulent while, on the other hand, as reported by other authors [18], WNV pathogenesis may be highly dependent on both the infecting strain and the avian host species.

Regarding the inflammatory response in the CNS, microglial cells were the most reactive population in the CNS of the WNV-infected partridges, mainly at 6 dpi (corresponding with the highest viral load), when most of these cells changed to an activated amoeboid morphology. The other resident immune effector of the CNS, astrocytes, appeared to play a limited role. Some authors have indicated that astrocytes can react later than the microglia and that their maximum activity occurs 14 days after CNS injury [42]. Therefore, it is possible that a more marked astrocytosis and/or astrogliosis would be detected later if our experimental study was extended. Microglia and astrocytes are considered essential in the anti-flavivirus response in the CNS of humans and rodent models [30,43,44,45,46]. In response to resident cell activation, among other stimuli, CD3+ T cells infiltrated the brain, especially at 6 dpi, and mainly in the cerebral pallium and molecular layer of the cerebellum. Upon antigen presentation, T cells act by directly destroying virus-infected cells and by producing cytokines that increase immune cell recruitment and stimulate other immune effectors [47]. In humans and rodents, it has been demonstrated that these cells are essential for WNV clearance and for recovery from the disease [43,48,49,50].

For a global interpretation of the WNV-induced encephalitis in the partridges, it is important to highlight that despite the essential defense role of all these cell components for the recovery from WNV infection; it has been indicated that a robust response can also have detrimental effects, contributing to neuron damage [43,51].

Although similar infection dynamics were found in partridges infected with Morocco/2003 and Spain/2007 strains, some differences were noted related to the viral load, virus antigen distribution, and severity of microscopic lesions. The most striking differences were found in the CNS, where encephalitis and neuronal necrosis were more acute and severe in partridges infected with Morocco/2003. The pathogenesis of WNV infection depends mainly on viral and host factors, which determine the level of viral replication and the severity of the infection [30]. As the infected partridges were homogeneous in terms of age, history of rearing, and maintenance, immunologic status should have been very similar between groups. For this reason, the observed differences were most probably due to factors related to the WNV strain. Genetic changes, particularly those leading to amino acid substitutions, can modify the virulence of WNV strains, as well as other phenotypic traits such as host and/or mosquito competence [52,53,54,55]. The Morocco/2003 and Spain/2007 WNV strains differ in 13 amino acid positions; therefore, it is possible that one or several of these changes determined/modulated the virulence of both strains for the red-legged partridge [21].

The pathogenesis of the WNV infection in the CNS depends mainly on the capacity of the virus strain to enter the CNS (neuroinvasiveness) and to produce lesions (neurovirulence) [56]. Some authors have indicated that, once a certain viremia level is reached, the ability of a WNV strain to enter the CNS is much more important than its own neurovirulence [57,58]. Although the mean peak viremia titer was higher in the partridges inoculated with Morocco/2003, there were no statistically significant differences between groups [17], and in both, WNV was present in the brain as early as 3 dpi. At 6 dpi, despite a similar viral load in the brain, a more intense inflammatory reaction and more severe microscopic lesions were observed in Morocco/2003-infected partridges. For these reasons, our results point out that differences in intrinsic neurovirulence between strains were more important than their neuroinvasiveness. It seems plausible that a higher neurovirulence of Morocco/2003, associated with a more exacerbated inflammatory reaction, resulted in a more acute and severe pathology in the CNS. Nevertheless, the exact mechanism by which this strain results in being more neurovirulent remains unknown, and the limited number of birds analyzed in this study forces us to be cautious with the interpretation of the results obtained.

In conclusion, the higher virulence of Morocco/2003 for the red-legged partridge is probably related to a more acute and severe encephalitis in the infected birds. Further studies are needed to elucidate the specific genetic markers that determine this higher neurovirulence.

## 4. Materials and Methods

### 4.1. Viruses

Two different WNV isolates from the Mediterranean basin were used in this study: Morocco/2003 (strain 04.05, GenBank acc. n°: AY701413) [20] and Spain/2007 (strain GE-1b/B, GenBank acc. n°: FJ766331) [21]. Details on the preparation of the inocula are given in Sotelo et al. [17].

### 4.2. Experimental Infection

The partridges were obtained, maintained, handled, and inoculated as described in Sotelo et al. [17]. Briefly, two groups of 7-week-old red-legged partridges were subcutaneously inoculated in the cervical region with 10^4^ PFU/individual of either WNV Morocco/2003 or Spain/2007 diluted in up to 0.1 mL Dulbecco’s Minimum Essential Medium (DMEM) (supplemented with 2 mM L-glutamine, 100 U/mL penicillin, and 100 μg/mL streptomycin). For the specific purpose of the present study, we performed euthanasia by intravenous injection of embutramide (T61^®^, Intervet–Schering-Plough, Madrid, Spain) on two birds of each group at days 3 and 6 post-inoculation (dpi), and of two birds infected with Spain/2007 and one infected with Morocco/2003 at 14 dpi.

### 4.3. Sample Collection

Detailed necropsies were performed on the euthanized individuals. Samples of the brain, heart, lung, liver, spleen, kidney, thymus, bursa of Fabricius, and feather pulp were collected into sterile polypropylene tubes filled with 1 mL of Hanks’ balanced solution (10% glycerol, 200 U/mL penicillin, 200 μg/mL streptomycin, 100 U/mL polymixin B sulphate, 250 μg/mL gentamicin, and 50 U/mL nystatin) and stored at −70 ºC until analysis by real-time reverse transcription polymerase chain reaction (RRT-PCR). In addition, samples of the brain, oral mucosa, thymus, heart, trachea, lung, liver, spleen, kidney, small and large intestine, pancreas, cecal tonsils, bursa of Fabricius, pectoral muscle, and skin with feather follicles were fixed in 10% neutral buffered formalin.

### 4.4. Virus Genome Detection

RNA was extracted from tissue samples after homogenization and tested by RRT-PCR for the presence of the WNV genome as described in Sotelo et al. [17].

### 4.5. Histopathology

Formalin-fixed tissue samples were trimmed, embedded in paraffin, and processed to obtain hematoxylin and eosin-stained sections. These were independently examined by two different investigators (UH and VG) to determine the presence of WNV-associated lesions. When lesions were present, they were graded according to their distribution (focal, multifocal, or diffuse) and severity (mild, moderate, or marked).

### 4.6. Immunohistochemistry

Tissue sections were mounted on Vectabond™ reagent (Vector Laboratories, Inc., Burlingame, CA, USA)-pretreated slides. Immunohistochemical detection of the WNV antigen was performed using a rabbit polyclonal antibody (BioReliance, Product 81–015, Rockville, MD, USA) at a dilution of 1:1000, following the protocol described previously [59]. We also characterized the inflammatory cell population in the cerebrum and cerebellum. For that purpose, we used primary antibodies, reagents, and protocols detailed in Table 4. Endogenous peroxidase activity was inhibited with a peroxidase-blocking reagent (Dako EnVision^®^+System-HRP (AEC), DakoCytomation, Carpinteria, CA, USA) (CD3, GFAP) or with 3% H202 diluted in methanol (RCA-1), rinses were performed using 0.1% Tris-buffered saline/Tween20 (TBS 0.05 M, pH 7.5), unspecific primary antibody labeling was blocked with 2% albumin from bovine serum (BSA) (Sigma-Aldrich Chemie, Steinheim, Germany) diluted in 0.1% TBS/Tween20, and sections were counterstained with Mayer’s hematoxylin.

Tissue sections of WNV RRT-PCR-positive red-legged partridges were used as positive controls. Controls of specificity included several sections with substitution of the primary antibody by 2% BSA−0.1% TBS/Tween20 and negative rabbit antibody (BioReliance, Product 81–015), and tissue sections of a non-infected (WNV RRT-PCR-negative) red-legged partridge (from sham-inoculated control group) [17]. For the detection of T cells, the positive control included a section of spleen of non-infected red-legged partridges. Negative controls included substitution of the primary antibody by 2% BSA−0.1% TBS/Tween20 and a brain section of a WNV RRT-PCR-negative partridge. A brain section of a non-WNV-infected partridge of the same age (control group [16]) served as reference for RCA-1 and GFAP.

We scored virus antigen staining according to its distribution and abundance in the tissues. The distribution of inflammatory cells within the brain was evaluated at 200x magnification. To detect changes in the abundance of CD3+ T cells and RCA-1+ cells during the course of infection, we counted the number of stained cells in 30 randomly selected fields at 400x magnification (in each brain region: cerebrum and cerebellum). Changes in the morphology, abundance, and staining intensity of GFAP+ astrocytes were also evaluated.

## Figures and Tables

**Table 1:** Viral genome load in tissues of experimentally WNV-infected red-legged partridges. The presence of WNV genome was analyzed in different tissues at different days post-inoculation (dpi) by real-time reverse transcription polymerase chain reaction (RRT-PCR). Data are presented as Ct values. SP07: Spain/2007, MO03: Morocco/2003, NA: tissue sample not analyzed, −: tissue sample with Ct ≥ 40.

	3 dpi				6 dpi				14 dpi		
	SP07		MO03		SP07		MO03		SP07		MO03
Tissue	No. 1	No. 2	No. 3	No. 4	No. 5	No. 6	No. 7	No. 8	No. 9 *	No. 10	No. 11
Brain	30.3	32.8	31.4	33.9	25.2	26.5	25.2	24.8	NA	−	37.6
Heart	28.7	28.5	25.4	24.8	19.6	20.3	23.7	18.8	NA	38.0	33.8
Lung	NA	30.9	31.0	30.1	26.3	25.7	29.2	24.2	NA	−	38.8
Liver	NA	30.2	29.6	30.9	28.7	25.7	30.8	28.2	NA	−	−
Spleen	25.3	23.7	26.5	24.4	29.7	22.7	24.6	21.8	NA	36.1	34.0
Kidney	26.3	27.5	24.5	26.0	23.0	22.4	23.7	20.9	NA	37.5	36.1
Thymus	27.6	29.3	31.9	23.5	30.2	23.4	33.0	NA	NA	−	29.0
Bursa of Fabricius	NA	32.0	32.8	34.2	27.9	24.8	31.5	29.8	NA	−	31.3
Feather pulp	−	31.4	27.4	31.3	18.2	20.8	22.8	16.9	NA	34.6	33.7

* Tissues from individual No. 9 were not available for analysis by RRT-PCR; however, they were used for histopathology and immunohistochemistry analyses.

**Table 2:** Microscopic lesions in tissues of experimentally WNV-infected red-legged partridges. Lesions were graded according to their distribution and severity at different days post-inoculation (dpi) in both groups, Spain/2007 (SP07) and Morocco/2003 (MO03). −: no lesion, +: focal and mild or moderate/multifocal and mild, ++: focal and marked/multifocal and moderate/diffuse and mild, +++: multifocal and marked/diffuse and moderate or marked. NA: tissue sample not analyzed.

	**3 dpi**				**6 dpi**				**14 dpi**		
	**SP07**		**MO03**		**SP07**		**MO03**		**SP07**		**MO03**
Tissue/Lesion	No. 1	No. 2	No. 3	No. 4	No. 5	No. 6	No. 7	No. 8	No. 9	No. 10	No. 11
**Cerebrum**											
Neuronal necrosis	−	−	−	−	−	−	+	+	+	−	+
Gliosis	−	−	−	−	−	+	+	++	+	+	−
Perivascular cuffing	−	−	−	−	−	−	−	−	−	+	+
Endothelial cell swelling	+	+	++	++	+	+	+	++	++	+	+
**Cerebellum**											
Purkinje cell necrosis	−	−	−	−	−	−	++	+	+	NA	+
Gliosis	−	−	−	−	−	−	++	+	−	NA	++
Perivascular cuffing	−	−	−	−	−	−	−	−	+	NA	−
Endothelial cell swelling	−	−	++	++	+	+	+++	++	++	NA	++
**Heart**											
Myofiber necrosis-degeneration	+	+	−	+	+++	++	+++	+++	++	+	++
Inflammatory infiltrate	−	++	−	++	+++	+++	+++	+++	+	++	+++
**Lung**											
Inflammatory infiltrate	−	+++	−	++	+++	+++	+++	+++	+	−	+++
**Liver**											
Hepatocyte necrosis	−	−	−	−	−	+++	−	+++	−	−	−
Inflammatory infiltrate	−	++	++	++	++	++	++	+++	−	++	+
Hemosiderosis	−	−	−	−	+	+	++	++	++	+	+
Tissue/Lesion	No. 1	No. 2	No. 3	No. 4	No. 5	No. 6	No. 7	No. 8	No. 9	No. 10	No. 11
**Spleen**											
Lymphoid cell necrosis	−	−	−	−	−	−	−	−	++	−	−
Lymphoid cell depletion	−	−	++	+	+	+	+	+	−	−	−
Granulocytic infiltrate	+++	+++	++	++	+	+++	−	−	+++	++	+++
Eosinophilic material deposits	−	−	−	−	+++	+++	++	++	++	−	++
Hemosiderosis	−	−	−	−	+++	+++	+	+++	++	−	+
**Kidney**											
Tubular epithelial cell necrosis	−	−	−	−	−	−	−	−	+++	+++	+++
Inflammatory infiltrate	−	++	−	++	−	−	−	+	+	−	++
**Duodenum**											
Inflammatory infiltrate	+	+	−	−	−	+	−	−	NA	−	+
**Large intestine**											
Inflammatory infiltrate	−	+	−	++	−	−	−	−	NA	−	++
**Cecal tonsils**											
Lymphoid cell necrosis	NA	NA	NA	+	NA	++	NA	NA	NA	−	NA
**Bursa of Fabricius**											
Lymphoid cell necrosis	−	−	−	−	NA	−	−	++	NA	NA	−
Lymphoid cell depletion	−	+	−	−	NA	++	++	++	NA	NA	−
**Skin + feather follicle**											
Inflammatory infiltrate skin	−	NA	−	+++	−	NA	NA	−	NA	++	−
Inflammatory infiltrate feather pulp	+++	NA	+++	NA	NA	NA	NA	+++	NA	−	+++

**Table 3:** WNV antigen detection in tissues of experimentally WNV-infected red-legged partridges. WNV antigen was detected by immunohistochemistry at different days post-inoculation (dpi) in both groups, Spain/2007 (SP07) and Morocco/2003 (MO03). Immunostaining was graded according to its distribution and percentage of stained cells. −: no staining, ±: focal single cells, +: focal or multifocal and <20% cells stained, ++: multifocal or diffuse and 20–50% cells stained, +++: multifocal or diffuse and >50% cells stained. NA: tissue sample not analyzed.

	3 dpi				6 dpi				14 dpi		
	SP07		MO03		SP07		MO03		SP07		MO03
Tissue	No. 1	No. 2	No. 3	No. 4	No. 5	No. 6	No. 7	No. 8	No. 9	No. 10	No. 11
Heart	±	−	+	+	++	++	+	+++	−	−	−
Lung	−	−	−	−	−	±	−	+	−	−	−
Liver	−	−	−	−	−	−	−	+	−	−	−
Spleen	+	+	+	±	−	+	+	+	−	−	−
Kidney	−	−	+	+	+	+	+	++	−	−	−
Duodenum	−	−	−	−	−	−	−	+	NA	−	−
Large intestine	−	−	−	−	+	+	+	+	NA	−	−
Pancreas	−	−	±	±	+	+	+	+	NA	−	−
Cecal tonsils	NA	NA	NA	+	NA	−	NA	NA	NA	−	NA

**Table 4:** Reagents and protocols used to characterize inflammatory cells in the brain of experimentally WNV-infected red-legged partridges.

Primary Antibody ^a^	Cell Population	Pretreatment ^b^	Primary Antibody Dilution and Incubation ^c^	Secondary Antibody ^d^	Detection System ^e^
Lectin RCA-1 biotinylated	Microglia-macrophages	Citrate buffer Microwave heat (22 min)	1:600, 45 min RT	Goat anti-rabbit IgG	ABC-DAB
Polyclonal rabbit anti-GFAP	Astrocytes	Proteinase K (7 min RT)	1:500, 4 °C ON	Labelled polymer-HRP anti-rabbit	AEC + substrate chromogen
Polyclonal rabbit anti-human CD3	T cells	Citrate buffer Microwave heat (22 min)	1:500, 4 °C ON	Labelled polymer-HRP anti-rabbit	AEC + substrate chromogen

^a^ Primary antibody products: RCA-1 product No. B-1085 (Vector Laboratories); GFAP product No. Z0334 (DakoCytomation, Glostrup, Denmark); CD3 product No. A0452 (DakoCytomation); CD79a product No. RM-9118 (Thermo Fisher Scientific, Runcorn, UK). ^b^ Proteinase K (DakoCytomation); RT: room temperature (22–25 °C). ^c^ Antibodies were diluted in 2% BSA−0.1% TBS/Tween20. ON: overnight. ^d^ Goat anti-rabbit IgG (Vector Laboratories) was diluted 1:200 in 0.1% TBS/Tween20 and applied for 1 h at RT; Labelled polymer-HRP anti-rabbit (Dako EnVision^®^+System-HRP (AEC), DakoCytomation) was applied according to manufacturer’s recommendation. ^e^ Avidin-biotinylated enzyme complex (ABC system, Vector Laboratories) was applied for 30 min according to the manufacturer’s recommendation and 3,3’-diaminobenzidine tetrahydrochloride (DAB, Vector Laboratories) was applied for 30 s according to the manufacturer’s recommendations. AEC+substrate chromogen (Dako EnVision^®^+System-HRP (AEC), DakoCytomation) was applied for 15 min (CD3, CD79) and 3 min (GFAP) according to the manufacturer’s recommendations.

## Data Availability

The data presented in this study are available in the different tables of this article.
